# Talking about the institutional complexity of the integrated rehabilitation system—the importance of coordination

**DOI:** 10.5334/ijic.851

**Published:** 2013-03-22

**Authors:** Sari Miettinen, Ulla Ashorn, Juhani Lehto

**Affiliations:** HAMK University of Applied Sciences, 13101 Hämeenlinna, Finland; School of Medicine, 33014 University of Tampere, Finland; School of Health Sciences, 33014 University of Tampere, Finland

**Keywords:** rehabilitation, welfare services, coordination, governance, equity, complexity

## Abstract

Rehabilitation in Finland is a good example of functions divided among several welfare sectors, such as health services and social services. The rehabilitation system in Finland is a complex one and there have been many efforts to create a coordinated entity. The purpose of this study is to open up a complex welfare system at the upper policy level and to understand the meaning of coordination at the level of service delivery. We shed light in particular on the national rehabilitation policy in Finland and how the policy has tried to overcome the negative effects of institutional complexity.

In this study we used qualitative content analysis and frame analysis. As a result we identified four different welfare state frames with distinct features of policy problems, policy alternatives and institutional failure.

The rehabilitation policy in Finland seems to be divided into different components which may cause problems at the level of service delivery and thus in the integration of services. Bringing these components together could at policy level enable a shared view of the rights of different population groups, effective management of integration at the level of service delivery and also an opportunity for change throughout the rehabilitation system.

## Introduction

The Finnish institutional system of rehabilitation policies has been presented as an example of a complex welfare system with several problems [1, 2]. In many other European countries rehabilitation is a complex part of the welfare system with quite similar problems [3–5]. However, in other countries rehabilitation policies are usually studied and discussed through concepts of disability or disability policies [6, 7].

Both national and international studies on the Finnish rehabilitation system have acknowledged several problems. The OECD has conducted a broad study on disability service systems in several countries where they reported that the Finnish rehabilitation system is a complex one with several subsystems and lack of coordination [3]. A recent report by the National Institute of Health and Welfare described the rehabilitation system as diffuse and fragmented [8]. Lack of cooperation especially has been mentioned as a key concern in national reports and studies of the system [9, 10]. Better cooperation between the subsystems has also been one of the main goals in reforming the system in recent decades [9, 11, 12]. These problems at the upper level of the system may lead to important problems at the level of service delivery, such as citizens falling through “the rehabilitation net” and ending up living without the rehabilitation services they need. Regarding international studies, Schmollinger [13] has also noted similar problems pertinent to the rehabilitation system in Germany. He pointed out that the system in Germany is also a combination of several kinds of services with a lack of clear cooperation and clear allocation of responsibilities.

What is needed in complex systems is an appropriate approach to achieve successful policy implementation [14]. One of the approaches acknowledged in earlier research is cooperation at the systemic level needed, such as adaptive co-management or collaborative work between different agencies [14, 15]. Jones [14] has also stressed the need to “remove the barriers to self-organisation”. These barriers could prevent different actors in the system “from adapting to emerging problems”. The need for cooperation is obvious in the rehabilitation system in Finland. However, the different subsystems are decidedly self-organised, which could raise coordination as one significant goal of governance [16]. With closer coordination it is possible to build bridges between different subsystems or institutions in order to provide better support for citizens at the level of service delivery [17]. Thus coordination and governance at system level could also lead to better integration at service delivery level.

At the higher organisational level of care systems the concepts of governance and coordination are used more in the social science and political science literature than the concept of integration [18, 19]. Definitions of integration vary and the concept is used in studies of both micro and macro levels of the social and health care system [see 17, 20]. To avoid confusion with the meaning of integration it could be useful to operate with different concepts at the higher organisational level. This is why we use coordination and governance to describe the micro level of the rehabilitation system in this article.

The Finnish institutional system of rehabilitation has numerous functions and is linked in several different welfare systems. In this article these linked welfare systems are referred to as subsystems. The various rehabilitation functions in Finland are: the provision function, the financing function, the social rights function, the steering function, the income compensation function and the professions function. The subsystems are: (1) decentralized health and social care service system, (2) active employment services, (3) obligatory sickness insurance and first pillar (basic) pension system (the public Social Insurance Institution, SII), (4) obligatory second pillar (earnings related) pension system, (5) obligatory traffic and work accident insurance systems (private insurance companies and private voluntary insurance systems) and (6) voluntary accident insurance system. Thus the subsystems are part of different welfare policies, such as social and health care and pension policies and the functions are usually grouped under medical, vocational and social rehabilitation [1]. In this complex system, a lack of common coordination may result in different population groups having different rights to rehabilitation services [21, 22].

One of the major goals in Finnish rehabilitation policies has been to create a more coordinated entity from the different and rather separate subsystems [9, 11, 12]. In general, various changes in Finnish society, such as the changing demands for the functional and social quality of the workforce have given rise to a need to rethink, reorganize and coordinate rehabilitation activities [1].

Several barriers to integration and also to coordination could be found in complex systems. One of the barriers is a lack of communication between different parts of the system [4, 23]. However, different ways to tackle the barriers are proposed, such as setting common goals between subsystems, institutions or organisations. For instance, if the goals vary, management may be ineffective and the foundation for integration is not perhaps stable enough [24, 25]. Overall, a common vision and goals at the macro-level of the system seems important to support efforts at the micro level [20].

Studies of complex combinations of rehabilitation systems and their population-wide policies are marginal on a national and also on an international scale [22, 26]. Although it is possible to find several studies on rehabilitation, they usually cover only a specific field such as medical or vocational rehabilitation [27–29]. In this article we will essentially contribute to the research field and open up the wide rehabilitation policies in order to understand this kind of complexity and problems related to it. Overall, rehabilitation has not been extensively focused on in the literature on integrated care, although rehabilitation could serve extensively as a good example of mixed welfare services and the problems of coordination and integration [13, 30, 31]. In this article, our focus is specifically on rehabilitation as a combination of different subsystems and not the integration of rehabilitation into other services such as health care services.

The purpose of this study is to open up a complex welfare system at upper policy level and to understand the implications of coordination for the level of service delivery. We will in particular address the policies to overcome the negative effects of the institutional complexity of rehabilitation policy in Finland. We also address the influence on different rights to rehabilitation services for different population groups. In order to understand the context of our study, our first question is: how has the complexity in rehabilitation policies developed in Finland? Our second question is: what kinds of frames were used in the discussions about developing the rehabilitation system in Finland? Overall, this study focuses on the coordination of a complex system at policy level. As our approach we use governance theories rather than organization theories. This study is part of a larger research project (during 2005–2007) studying rehabilitation system in Finland from different perspectives [1, 32].

### Historical roots of the complexity of rehabilitation policy in Finland

The first observation regarding the history of rehabilitation services is that there is no history of rehabilitation as such, because there was never a shared concept of the practices and policies that are currently grouped under the concept of rehabilitation. The beginning of the broad use of a single Finnish concept of rehabilitation (in Finnish *kuntoutus*) can be traced back to the 1960s and 1970s [33–35]. However, it was still clearly an artificial concept. Additionally, the borders between different rehabilitation functions (medical, social or vocational) or between the social rights of the beneficiaries of the different rehabilitation systems became increasingly confusing causing many kinds of conflicts [21].

A second observation on the history is that what is today understood as rehabilitation has mainly developed from a rather marginal part of social and health policies, such as social assistance for people with disabilities, disability pensions, traffic and work accident insurance, active labour market policies, education policies to return dropouts from the labour market to employment and also health promotion, primary health care and aftercare of hospital patients. Thus, the rehabilitation aspects of these welfare systems have rather reflected the general fundamental ideas of the different systems than any common idea of how rehabilitation policy should be institutionally organised and implemented or coordinated.

There have been functions of different welfare systems that from the present perspective could also be called rehabilitation earlier in history. However, the history of building up a rehabilitation system in Finland often starts from the broad medical and vocational rehabilitation activities developed to restore the work or functional capacity of those injured during World War II. Based on the Welfare and Care of Invalids Act, rehabilitation was organised on government funding in the late 1940s as part of the local social assistance services [35].

The next layer of vocational and medical rehabilitation was built within the basic flat rate universal pension system passed by Parliament in 1956 [36] and the universal sickness insurance system enacted in 1963 [37] (first pillar pension system in Table 1). Both were universal systems run by the public Pension and Sickness Insurance Fund. The old legislation on traffic accident and work accident was also amended. The purpose was to provide, among other things, universal rehabilitation coverage after work- and traffic-related accidents [35, 38]. In the 1960s the notion of social rehabilitation also gained strength [39]. However, while funding was rather meagre and rights were universal, at that time the actual coverage of rehabilitation activities was rather limited. A limited coverage of rehabilitation activities, particularly those other than related to war injuries, before the 1970s, can be explained by the late development of modern welfare state in Finland. The development of sickness insurance, in the late 1960s, of tax funded public primary health care in the 1970s, of modern activation services and benefits for the unemployed as well as social care services, particularly in the 1980s, all included rehabilitation among their range of activities [35, 39, 40].

The Welfare and Care of Invalids Act was fused into the municipal social and health care service system in 1987. Overall, the new policies from the 1960s to the 1980s included a rehabilitation component (medical, social, vocational or a combination of these) in their service options and coverage [35, 40].

### Rehabilitation system as a mix of different financing schemes

The rehabilitation system in Finland is based on several financing schemes (Table 1). In the beginning, in the 1940s, the rebuilding of the nation after World War II was a national task and the financing was based on taxes and organized through the Social Ministry.

The Finnish welfare state expanded rapidly from the 1960s onwards and new institutional options emerged [40]. Economic growth offered more resources for the policy reforms and the ideology of the welfare state in Finland was supported by the labour market organizations. The number of unemployed people in Finland increased due to an exceptionally rapid transformation from an agrarian to an urbanized, industrialized and service economy country [38]. Thus the earlier competences of the workforce no longer met the new labour market demands. This was a challenge for all aspects of rehabilitation. In the 1960s the rather meagre (‘Bismarckian’) sickness insurance coverage was complemented by a tax funded and publicly provided (‘Beveridgean’) primary health service and hospitals, which today form the core health system, the sickness insurance funded private system being only a smaller complementary system [35, 38].

In the 1980s the old social assistance type of invalidity welfare was incorporated into the expanding universal Beveridgean service system of social care. This system was also closely linked to the Beveridgean component of the health system creating a more or less fused “local social and health care system”. In the 1970s and 1980s Finland also experienced an expansion of tax revenue funded active employment services [40, 42].

Since the 1980s different elements of the rehabilitation system have been perceived as parts of a whole, although they have also been developed separately. On the one hand, the elements were gathered under a single concept while on the other a government working group was established to clarify the role of the different elements from the perspective of the whole. Then the first coordinating body, the Advisory Board for Rehabilitation (KUNK), was set up with an advisory function towards the Ministry of Social Affairs and Health [43].

Over time, new parts were thus added to the system without dismantling the old institutions. This resulted in a complex institutional entity that includes the first pillar (basic public universal flat rate) and the second pillar (earnings-related labour market-based) insurance systems, obligatory traffic and work accident insurance (accident insurance offered by private companies) as well as Beveridgean tax revenue funded mainly public employment, social and health care services and a Bismarckian sector? of insurance funded private health care services. The different subsystems have also been based on different governing principles and structures. The pension subsystems are governed by tripartite bodies representing labour market parties and the government. By contrast, the accident insurance subsystems tend to follow a model of government controlled private company governance. The public employment services are run by local offices directly under central government while the local social and health care services are under municipal decision-making under relatively light central government guidance.

Several problems were acknowledged in the complex rehabilitation system and the problems were addressed especially in the 1970s and 1980s. One main problem mentioned in different national rehabilitation policy documents was absence of effective co-operation between the subsystems, which increased the risk of people falling through “the rehabilitation net” in the level of service delivery [44]. At the beginning of the 1980s the new Advisory Board for Rehabilitation had its first large mission. It had to prepare a major legislative reform to address the problems acknowledged in the rehabilitation system. After several years’ work, the reform was launched in 1991 and became the largest legislation reform of the rehabilitation system in Finland so far. There was also a keen interest in following the realisation of the new legislation. Thus, the Government was required to report to Parliament every fourth year on the realisation of the legislation and the overall developments in the field of rehabilitation in the form of a rehabilitation report. The first report was submitted in 1994 and the last in 2002. Since 2002, the same kind of evaluation of the rehabilitation policy and system has been included in the Government’s broader social and health reports [45]. However, in these reports the role of the rehabilitation system is much more marginal than in the earlier rehabilitation reports.

## Data, theory and methodology

### Data

The data in this article are derived from the last rehabilitation report of 2002 and related materials. This was the last report to focus on and describe the development of the rehabilitation system. The data consist specifically of detailed minutes of the plenary session of the parliamentary debate on the 2002 rehabilitation report. The complementary data are the main report [9] with its background documents and interviews with key authorities in the Finnish rehabilitation system. The data are presented in Table 2.

The main data are the detailed minutes of the parliamentary debate on the 2002 rehabilitation report. In all, the four rehabilitation reports are all an important part of rehabilitation policy in Finland. Around the rehabilitation reports a kind of a polity [46, 47] of the whole field of rehabilitation in Finland was created. In this polity, the various political actors made efforts to influence the governmental rehabilitation policy. The significant issues in this polity have been: 1) coordination of the actions of the different rehabilitation sectors and institutions at national, local and professional client work levels. 2) Evaluation of the efficiency of rehabilitation and, consequently, the improvement of the efficiency of rehabilitation activities.

To understand the significance of the plenary session to the rehabilitation policy in Finland the parliamentary process of the rehabilitation reports needs to be discussed. After being presented to Parliament, the reports went through a long parliamentary process. At first, Parliament had a long debate about the report in a plenary session. Every detail of the discussion was documented in the minutes. The discussion followed a specific pattern with every Parliamentary group presenting their own views on the report. The discussion is a significant and prominent part of rehabilitation polity in Finland. The documentation of the discussion is extensive, amounting to 49 pages. First the ministers concerned (in this case the Minister of Social Affairs and Health and the Minister of Health and Social Services) described the outline of the report. After that, every parliamentary group presented their own views on it. The discussion continued with the comments of individual MPs, answered by the appropriate ministers. In 2002 the parliamentary groups were formed by the same political parties as today: the Centre Party, the Finnish Social Democratic Party, the National Coalition Party, the Left Alliance Party, the Greens of Finland, the Swedish People’s Party, the Christian Democrats and the True Finns Party.

As discussed above, the rehabilitation report of 2002 and thus the plenary discussion thereon was the last in the history of rehabilitation reports in Finland. Although the report and the discussion were published years ago, there have so far been no major reforms of the whole rehabilitation system in Finland. According to the recent national policy reports the rehabilitation system still appears as complex as it was in 2002 [8]. Thus, these ten-year-old documents reflect the situation today and even the same problems emerge today as years ago.

The complementary data were the Rehabilitation Report of 2002, its background documents and interviews with key informants. The data were used to complement the findings from the primary data. At a concrete level the complementary data were used after the main findings had been made from the main data. The complementary data helped to understand the main data or elaborated the missing parts in the main data. The background documents included letters and statements from the time of the preparation of the Rehabilitation Report. We used several archives to find the needed papers. The authorities interviewed were from the Ministry of Social Affairs and Health, labour market organizations and the insurance system. In all, we interviewed five key authorities of the rehabilitation system individually for the study. We chose the authorities depending on the findings from the primary data. If we needed a more profound or wider perspective on some issues we chose a relevant authority to complete the picture of the rehabilitation system. It was important to discuss with the authorities because, for example, the insurance system was hardly touched upon in the plenary discussion although it is an important part of the rehabilitation system. All interviews also gave us a more recent perspective on the system.

### Theory and methodology

In this study we use the concept of coordination as a goal of governance. Governance is a concept often used to comprehend complex welfare systems and their problems [48–50]. One perspective on governance is to understand it as different coordination models, such as hierarchy, network or market [50, 51]. Exworthy et al. [52] have also highlighted the meaning of quasi-hierarchy, quasi-market and quasi-network as well as important coordination models. A recently coined concept is hybrid coordination, meaning different combinations of the individual coordination models [53]. In this study we used the different coordination models to understand the complexity of the rehabilitation system and its meaning for the integration of the services.

The analysis was twofold. The first part was data based concentrating on the detailed minutes. The second part was based on the literature.

The first part of the analysis was done using data based content analysis [54–56]. By this method we searched and categorised themes from the text. First we searched the detailed minutes for excerpts about the rehabilitation system as a whole instead of only separate subsystems. The word system here refers to the subsystems [1] and the Government of Finland. In our analysis government refers particularly to the state budget and the state administration including Parliament, the Cabinet, ministers, ministries and their special committees e.g. the Advisory Board for Rehabilitation. Basically, the excerpts identified described the positions of subsystems and the government in the field of rehabilitation, their strengths and weaknesses. After the identification of the excerpts we categorised and analysed the problems mentioned and the solutions proposed. This kind of division between problems and solutions is commonly used in policy analysis [57].

After the data based content analysis we identified different governing perspectives among the problems and solutions using frame analysis. We saw the frames as boundaries of different views on the rehabilitation system [58]. Concretely searched the text to find what kinds of frames or scenes were created during the definition of the problems and solutions. The frame analysis produced certain frames of interpretations of developing the system. Thus, the frames were the different ways of talking about governing. We also analysed the political division between the frames. We were interested in how the opinions of the MPs from different political parties were divided between the frames identified and whether different political parties favoured different frames.

In the second part of the analysis we interpreted the frames identified referring to the relevant literature on the welfare state and governance [e.g. 18, 51]. We were interested especially in the roles of the welfare state. This served to enhance the analysis and make it more theory oriented.

We checked and confirmed the results using the complementary data. The complementary data were analysed in the same way as the main data. The interviews were transcribed and thus in a written format. The interviews were thematic and conducted by two researchers. The interviews were planned in advance and certain themes such as “the rehabilitation system as a whole”, “development of the system” and “roles of different subsystems” were discussed with the key authorities. The interviews were audiotaped and transcribed and the texts were first analysed by one researcher. In the next phase the analysis and results were discussed with the research group and finally the results were processed together.

## Results

The discussion between the representatives in the plenary session was colourful. Clashes of opinion were easily discernible in the text. As a result we identified four different frames in the debate (Table 3).

In the first frame the main problems identified were inequality and inequity between individuals. The shared aim in this frame was universality and also equity of rehabilitation services. However, these aims were somewhat unsuccessful. We interpreted the institutional failure as fragmentation within and between institutions, especially financiers and providers of rehabilitation services. Failure was addressed, for instance, with regard to the pension institutions and the state administration. In all, this was a traditional welfare state frame where the aim of equality and equity between population groups resembled the notion of universality.

In the second frame we identified two perspectives on the problems. First, the workforce was retiring too early. Without the tax revenue, less government money would be available for the services in the future. Second, the level of well-being and health among workers was too low. Thus it seemed that the institutions had only partially met the needs of society for rehabilitation. The focus was to extend the overall career length and address the need better. In all, the perspective of this frame was a competition state, especially its corporatist component, because in this context the trade unions exert considerable influence.

In the third frame the main problems were wasteful use of resources, unfair distribution between payers and beneficiaries and lack of evaluation of the cost-effectiveness of rehabilitation. In addition to not having sufficient funds to finance the services, the services financed by the welfare state had been ineffective. We interpreted the institutional failure as a lack of discipline. For instance, some pension institutions were not paying enough for rehabilitation services although they received the benefit. In all, such a critical perspective of a wasteful and ineffective welfare state has long roots in welfare state debates.

In the fourth frame the main problem was unfair competition between rehabilitation service providers. This was said to undermine some rehabilitation services. Here we interpreted the institutional failure as fragmented markets. It seems that the semi-markets have created a situation giving rise to unfair competition between rehabilitation service providers. Certain features in some of the service provider organisations may have weakened their position in the competition. The fragmentation seemed to emerge especially between the financiers and providers of the rehabilitation services. In all, this was a clear competition state frame.

In different frames different policy solutions were proposed for the problems described. The solutions in the first frame were usually aimed at legislation. Several obligations were assigned to the institutions and the rights were correspondingly assigned to individuals. In the second frame legislation was called for to create new obligations for some institutions while increases in financial resources were proposed for the rehabilitation services. In the third frame the main solution was the allocation of funding on the basis of effectiveness. The aim was to “get value for money” from the expensive rehabilitation services. In the fourth frame, the MPs expected a solution, but the data provided no evidence of any suggestions on what these solutions might be.

After having identified the frames we also found it important to see how they were distributed among the political parties. As can be seen in Table 4, there were some differences in the political distribution. First, two frames permeated the whole political field. Second, the parties currently represented in the government tended to stress the reproduction of labour force. Third, the competition reflected and the fourth frame was closer to the competing service providers. These providers were represented by the National Coalition Party and the Centre Party.

## Discussion

Several perspectives on rehabilitation policy have been used in the same policy discussion to overcome the problems resulting from the institutional complexity of the rehabilitation system. The four frames represent four preliminary patterns of coordination and thus the complexity of governance in the system. The existence of four different frames is essential because the way a policy problem and solution are framed assigns responsibility and creates rationales that enable some policy solutions and impede others. For instance, policymakers need to commit themselves to the priorities between the frames and thus, between the subsystems. The different frames may also lead to a preference for diverse coordination models between the subsystems or institutions and thus different solutions in practice resulting to different rights for different population groups.

The different frames also highlight different thoughts on the coordination of this complex system. From one perspective the problem is missing networks, the other perspective emphasises the need for command and control and the last perspective focuses on ineffective and unfair markets. Thus, it is possible to find several different coordination models in the system which refers to some kind of hybrid coordination. Such aspects are important to address if the aim is to find an appropriate approach to policy implementation in this system of mixed coordination.

Overall, the results suggest that the nature of coordination is essential in complex systems. One finding is that in the case of rehabilitation the lack of common coordination at the system level may lead to a lack of successful integration at the level of rehabilitation services. In this case the successful integration of services is achieved when the continuity of a needed rehabilitation service or process is not interrupted although a person is moving between different subsystems and thus the subsystem or institution responsible is changed. Another finding is that, in order to understand the system, its coordination or integration, it is important to acknowledge the origin and background of the complexity.

It seems that within the different subsystems, different perspectives on rehabilitation policy have been sustained and largely resisted a stronger governance of the whole. While there have been efforts to create coordinating bodies such as the Advisory Board for Rehabilitation and thus create some kind of centralized coordination, the system has retained its institutionally complex characteristics to this day. Evidently the Advisory Board and the rehabilitation reports to Parliament have contributed to a shared understanding of the large complex system, but have not yet resulted in any strong governance of the whole. At the core of this governance effort are the parliamentary discussions about the rehabilitation reports. Despite the Advisory Board there seems to be no shared view about the governance [1]. From this perspective a major change in the whole system does not seem probable.

The institutional complexity of the rehabilitation system could partly be explained by its history. Until the 1960s coordination seemed simple. With the expansion and the ideological adoption of the Finnish welfare state since the 1960s, the idea of a working society grew stronger and new elements were added to the institutional structure of rehabilitation so that it covered as many disabled people as possible. At the same time the emphasis on paid work and the stigmatization of unemployment put pressure on disabled people. At the system level the increasing trend of policy reforms in Finland influenced the expansion of the system. It became complex with several subsystems, several coordination models and several views on how to develop this mixed welfare system, the whole of rehabilitation in Finland.

The history of this complexity has an important meaning through path dependence [59, 60]. This means that the history has created resistance to major changes in the rehabilitation system. One perspective on locking the path points to the different interest groups with different institutional positions [39]. Major changes such as joint legislation among different sectors and services are difficult to achieve because, for instance, the risks of change are perceived differently by different interest groups such as trade unions and municipal decision-makers. However, no such significant disagreements were found at the level of the political parties. There was only a minor division in the left-right alliances, which are strongly influenced by the trade unions (left) and competing rehabilitation service providers (right). Instead, discourses such as ineffectiveness and equality seemed to have permeated the whole political field.

The perspective on the history of the rehabilitation system in Finland underlines the difficulty of achieving effective cooperation between the subsystems or institutions in such a complex system. The subsystems are decidedly self-organised with meaningful histories and strong actors. Thus, the lack of a common understanding of the main goals in developing the systems is not surprising. What is needed is something that could break down the barriers between the strong self-organised subsystems.

Shared governance could open the way to changing the system. A shared vision, staying on the path or breaking the path, could be the key. At the time of the parliamentary discussion a shared vision seemed to be lacking. Even now the lack of a shared view will also challenge the development of any joint coordination of the rehabilitation system. However, this vision could be based on the present discussion about extending the age of retirement in Finland.

All things considered, it seems that, depending on which of the frames is emphasized, different population groups end up receiving rehabilitation services. If the system is developed using the frame of “reproduction of workforce”, the primary group to receive rehabilitation will perhaps be the working population. The justification is that effective rehabilitation will probably enable them to return to work. Similarly, if the emphasis of development is on equality, perhaps the retired population may also have a stronger claim to rehabilitation. With regard to these examples we may ask how it is possible to achieve effective integration between different services if the coordination at the macro-level is as fragmented as described.

## Conclusion

A shared view at the macro level of the complex system and its future seems important to gain a more coordinated system, also at the level of service delivery. Rehabilitation policy in Finland seems to be divided into at least two main components which should be brought together in order to gain a shared view. These parts are the perspective of the traditional welfare state with government led centralized solutions and that of the competition state with a corporatist and market oriented component. However, no indications of such a consensus were found in the plenary debate or mentioned in the interviews.

Another aim to gain a shared view could be to use a strong tripartite body to act as a coordinator as is the case in the pension system in Finland. But could strong corporatism compensate weaker government? In Finland the tripartite governance system of the government and the labour market representatives has been used in the governance of pension policies and in incomes policies. However, even with regard to these policy sectors, the tripartite governance model has met with increasing resistance [e.g. 61]. Thus, coordination based on the traditional tripartite system is perhaps not the one to build governance in the future.

All things considered, none of the options for a coordinating model for such a complex system as rehabilitation in Finland is an easy one. As long as common coordination is missing at the system level, the integration of different rehabilitation services at the service user level will be challenging and the rights of different population groups in this complexity will vary.

In order to achieve well-coordinated integrated services and smooth service processes “the black box” needs to be opened. It is important to understand the construction of the system. If the system is as fragmented as is the rehabilitation system in Finland with its strong history and independent subsystems, cooperation will be no easy task. Although cooperation has been one of the main tasks of Finnish rehabilitation policy at least since the 1990s, it has not removed the main problems from the system. In the rehabilitation system one way to approach the complexity could be to recognise its hybrid coordination model and the special features of each subsystem. These way appropriate tools could be found and common coordination could be achieved.

## Reviewers

**Jane Hendy**, Phd, Senior Lecturer in Health Management and Policy, Head of PhD programmes, The Department of Health Care Management & Policy, University of Surrey, Guildford, Surrey GU2 7XH, UK

**Simo Kokko**, Chief Medical Officer, Director of the Unit for Primary Health Care, Health Authority of Northern Savo, Finland; Adjunct professor, University of Eastern Finland, Kuopio, Finland

**Ulla Wihlman**, RPT, MSC, PhD. Senior Research and Evaluation Consultant, Stockholm, Sweden

## Figures and Tables

**Table 1.**
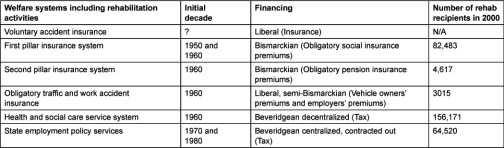
Initial decades, financing bodies and number of rehabilitation recipients of the rehabilitation subsystems in Finland. The latest official information about rehabilitation recipients is from 2000 [1, 9].

**Table 2.**
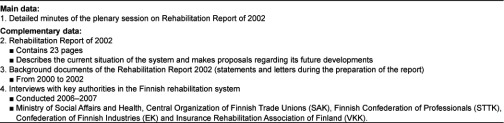
Data used in the study.

**Table 3.**
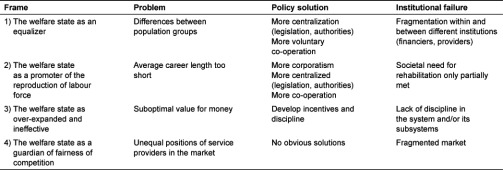
Characteristics of the frames.

**Table 4.**
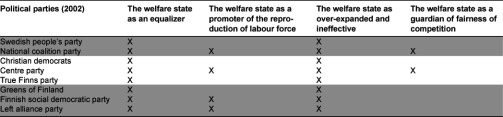
Frames used by the political parties. Parties represented in the coalition Government in 2002 are marked by dark shading. The symbol X indicates the frames favoured by the parties.
